# The role of sociodemographic factors associated with waterpipe smoking among male adolescents in western Iran: A cross-sectional study

**DOI:** 10.18332/tid/91601

**Published:** 2018-06-05

**Authors:** Saeed Bashirian, Majid Barati, Hamid Abasi, Manoj Sharma, Manoochehr Karami

**Affiliations:** 1 Social Determinants of Health Research Center, Hamadan University of Medical Sciences, Hamadan, Iran; 2 Department of Public Health, School of Health, Hamadan University of Medical Sciences, Hamadan, Iran; 3 Behavioral & Environmental Health School of Public Health, Jackson State University, Jackson, United States; 4 Research Center for Health Sciences, Hamadan University of Medical Sciences, Hamadan, Iran

**Keywords:** male adolescents, risk factors, student, water pipe smoking

## Abstract

**INTRODUCTION:**

Waterpipe smoking (WPS) is an increasingly popular leisure activity among young people in Iran. The purpose of this study was to identify the role of sociodemographic factors associated with WPS among male adolescents in Iran.

**METHODS:**

The study used a cross-sectional design. It included 730 high school male students (Grades 10–12) recruited through multistage random sampling conducted in 2017 in Hamadan city, western Iran. The self-administered questionnaires included information on demographic variables and behavioral risk factors related to WPS. Descriptive statistics and multinomial logistic regression modeling were conducted using SPSS.

**RESULTS:**

The student mean age, and standard deviation (SD), and age at WPS initiation were 16.41 (0.84) and 13.31 (2.43) years, respectively. The percentages of never, former and current WPS were 37.3%, 36.4% and 26.3%, respectively. We found that ever cigarette smoking (OR=5.14, 95% CI: 2.56–10.32) and WPS family (OR=2.55, 95% CI: 1.40–4.64) were significantly associated with former WPS. Furthermore, being 18 years, studying in technical fields, reporting ever and current smoking of cigarettes and family usage of WP were significantly associated with current WPS. Friends with WPS (OR= 0.50, 95% CI: 0.34–0.72) however played a protective role on former WPS.

**CONCLUSIONS:**

The results indicate that the prevalence of former and current WPS was high in Hamadan city. Thus, designing and implementing interventions for increasing students’, friends’ and family’s awareness regarding the harms of WPS and cigarette smoking are necessary to facilitate behavior change.

## INTRODUCTION

Waterpipe (i.e. hookah, narghile, hubble-bubble) smoking (WPS) has been an old tradition in different parts of the world^1^. Smoke for consumption is produced by burning wood coal situated at the top of a perforated aluminum foil below which mostly flavored tobacco blend is placed^2^.There are several harms associated with smoking waterpipe such as cancers, cardiovascular disease, stroke and others^3^. However, smoking waterpipes continues to be a common form of tobacco use in various regions of the world, particularly the Middle East. Many people, particularly in the Middle East, think that WPS is less harmful than cigarette smoking or even harmless^4^, but studies have shown that WPS has serious, chronic, harmful effects, particularly on the respiratory and cardiovascular systems^5^.

It is estimated that 100 million people, particularly teenagers, engage in WPS6. Since the 1990s, it has also become prevalent in the developed countries of the West^7^. Results from the 2010 Canadian Youth Smoking Survey^8^ revealed prevalence estimates of 10.1% for ever use and 4% for current use of WP among students in Grades 9–12. In a study in Iran, 9.7% of students smoked WP in the past month, of which 66.6% were male and 33.4% were female^7^. In another study in Iran, WPS prevalence was reported as 17.1% in male high school students^9^. WPS has a strong social aspect that makes it attractive and addictive to its users^10,11^. The results of a qualitative study showed that WPS is considered as enjoyable entertainment among friends, regardless of the health outcomes^12^.

Teenagers are especially attracted to the fragrance, to the pipe’s nice appearance, easy accessibility, low cost, less stigma, and greater social acceptance^7^. There is generally a paucity of studies done on WPS in Iran, especially among high schools^13,14^. Increasing prevalence in Iran and associated negative sequelae warrant planning and implementing preventative approaches that are geared for adolescents in particular. It is generally well accepted that epidemiological studies identifying the determinants of risky behaviors such as WPS, should be undertaken before developing interventions^15^.Therefore, the purpose of this study was to estimate the prevalence of WPS and assess behavioral and social predictors of WPS among male adolescents in Iran.

## METHODS

### Study participants

A total of 780 participants were recruited in the study. However, only 730 participants finished the study yielding a 93.5% completion rate. Multistage random sampling from 13 schools in two sectors of Hamadan city was employed with 60 students from Grades 10–12 participating in the study from each school. This study was approved by the Ethics Committee of Hamadan University of Medical Sciences (ID: IR.UMSHA.REC.1396.21). Informed consent, after due explanation, was obtained from all participants before the commencement of the study.

### Measures

The instrument was a self-report and consisted of information on demographic variables and WPS-related behaviors. The completion of the instrument took about 15–20 minutes. The instrument was divided into two parts: 1) Demographic factors including age, grade, major, father’s and mother’s occupation, father’s and mother’s education, whether having own room and living status; 2) WPS-related behaviors including being never, former (smoked waterpipe even one or two inhalations in your life) and current (smoked waterpipe at least once in the previous month) WP smoker, age at WPS initiation, computer games (at all, low, medium, high) internet usage (at all, low, medium, high)^15^, experienced cigarette smoking (yes/no), current cigarette smoking (yes/no), having friends who smoke WP (yes/no), and having family members who smoke WP (yes/no).

### Data analysis

Descriptive statistics including frequency, percentage, mean and standard deviation were used to describe study participants. To determine prevalence and predictive factors, a series of analytical tests and multinomial logistic regression were done. Adjusted odds ratios (AORs), with associated 95% confidence intervals (CI) are reported. All statistical analyses were performed using version SPSS 22.0. P-values less than 0.05 were considered statistically significant.

## RESULTS

In this study students’ ages ranged from 15 to 18 years with a mean of 16.41±0.84 years. A total of 324 (44.4%) students were from Grade 1, while 272 (37.3%) were from Grade 2 and 134 (18.4%) were from Grade 3, at high school level. Regarding the educational status, 231 (31.6%) students were in natural sciences, 201 (27.5%) in technical fields, 168 (23.0%) were in human sciences and 130 (17.8%) in mathematical sciences. Most students, 228 (31.2%), used a computer, and a total of 294 (40.3%) used Internet for more than 7 hours. Table 1 presents demographic characteristics of the students.

**Table 1:** Demographic characteristics of the study participants (n=730)

*Variables*	*Categories*	*Number (%)*
**Age (years)**	15	96 (13.2)
	16	310 (42.5)
	17	253 (34.7)
	18	71 (9.7)
**Major**	Human sciences	168 (23.0)
	Natural sciences	231 (31.6)
	Mathematics	130 (17.8)
	Technical	201 (27.5)
**High school grade**	Tenth	324 (44.4)
	Eleventh	272 (37.3)
	Twelfth	134 (18.4)
**Father's education**	Illiteracy	129 (17.7)
	Under the diploma	225 (30.8)
	Diploma	334 (45.8)
	College	42 (5.8)
**Mother's education**	Illiteracy	100 (13.7)
	Under the diploma	211 (28.9)
	Diploma	362 (49.6)
	College	57 (7.8)
**Father’s occupation**	Unemployed	75 (10.3)
	Worker	148 (20.3)
	Self-employed	387 (53.0)
	Employee	101 (13.8)
	Retired	19 (2.6)
**Mother’s occupation**	Housewife	59 (8.1)
	Employed	671 (91.9)
**Having own room**	Yes	537 (73.6)
	No	193 (26.4)
**Living (with)**	Both parents	693 (94.9)
	Others	37 (5.1)

According to WPS behaviors, 272 (37.3%) were never WP smokers, 266 (36.4%) former WP smokers and 192 (26.3%) current WP smokers. The mean (±SD) age at WPS initiation was 13.31±2.43 years. A total of 531 (72.7%) students had never experienced cigarette smoking. A total of 403 (55.2%) students’ friends did not smoke WP. Table 2 presents behavioral risk factors relating to WPS among the students.

**Table 2:** Behavioral risk factors characteristics among students (n=730)

*Variables*	*Categories*	*Number (%)*
**WPS**	Never	272 (37.3)
	Former	266 (36.4)
	Current	192 (26.3)
**Work with computer**	At all	228 (31.2)
	Low (1–3 h/d)	226 (31.0)
	Medium (3–7 h/d)	159 (21.8)
	High (>7 h/d)	117 (16.0)
**Internet usage**	At all	75 (10.3)
	Low (1–3 h/d)	172 (23.6)
	Medium (3–7 h/d)	189 (25.9)
	High (>7 h/d)	294 (40.3)
**Ever cigarette smoking**	Yes	199 (27.3)
		
	No	531 (72.7)
**Current cigarette smoking**	Yes	97 (13.3)
		
	No	633 (86.7)
**WP user friends**	Yes	327 (44.8)
	No	403 (55.2)
**WP user family**	Yes	117 (16.0)
	No	613 (84.0)

WP: waterpipe, WPS: waterpipe smoking

Table 3 shows that being 17 years old (AOR=3.61, 95% CI: 1.53–8.52), 18 years of age (AOR=3.99, 95% CI: 1.28–12.40), and studying in the technical fields (AOR=2.70, 95% CI: 1.49–4.90), were statistically significant predictors for current WPS. There was a trend (with reference group: 15 years old that never smoked WP) for increased likelihood of current smoking WP with increasing age; odds were 3.61 times higher for 17 years old (p=0.003) and 3.99 times higher for 18 years old (p=0.017) than for the reference group. Studying in the technical fields had odds of current WPS that were 2.70 higher than students in human sciences (p=0.001).

**Table 3:** Multinomial logistic regression: WPS and demographic factors (n=730)

*Characteristics*	*Never WPS (n=272) (%)*	*Former WPS (n=266) (%)*	*AOR ( 95% CI)*	*p*	*Current WPS (n=192) (%)*	*AOR ( 95% CI)*	*p*
**Age^a^ (years)**				0.462			0.718
15^b^	43 (5.9)	35 (4.8)	1.00	-	18 (2.5)	1.00	-
16	127 (17.4)	110 (15.1)	1.00 (0.58–1.73)	0.988	73 (10)	1.40 (0.72–2.74)	0.315
17	77 (10.5)	96 (13.2)	1.68 (0.81–3.48)	0.164	80 (11)	3.61 (1.53–8.52)	0.003**
18	25 (3.4)	25 (3.4)	1.84 (0.67–5.02)	0.236	21 (2.9)	3.99 (1.28–12.40)	0.017*
**Major^a^**							
Human sciences^b^	64 (8.8)	72 (9.9)	1.00	-	32 (4.4)	1.00	-
Natural sciences	92 (12.6)	92 (12.6)	0.69 (0.41–1.17)	0.172	47 (6.4)	0.68 (0.36–1.30)	0.248
Mathematics	53 (7.3)	48 (6.6)	0.60(0.33–1.12)	0.112	29 (4)	0.60(0.29–1.25)	0.178
Technical	63(8.6)	54(7.4)	0.64(0.37–1.09)	0.104	84 (11.5)	2.70(1.49–4.90)	0.001
**High school grade^a^**							
Tenth^b^	134 (18.4)	115 (15.8)	1.00	-	75 (10.3)	1.00	-
Eleventh	87 (11.9)	104 (14.2)	1.28(0.73–2.23)	0.385	81 (11.1)	1.02(0.55–1.90)	0.945
Twelfth	51 (7.0)	47 (6.4)	0.70(0.32–1.55)	0.386	36 (4.9)	0.82(0.34–1.99)	0.673
**Father's education^a^**							
Illiteracy^b^	12 (1.6)	16 (2.2)	1.00	-	14 (1.9)	1.00	-
Under the diploma	129 (17.7)	130 (17.8)	0.62 (0.25–1.50)	0.290	75 (10.3)	0.50 (0.19–1.29)	0.154
Diploma	80 (11.0)	80 (11)	0.70 (0.27–1.84)	0.475	65 (8.9)	0.84 (0.30–2.33)	0.736
College	51 (7.0)	40 (5.5)	0.58 (0.19–1.78)	0. 345	38 (5.2)	0.95 (0.29–3.13)	0.935
**Mother's education^a^**							
Illiteracy^b^	23 (3.2)	16 (2.2)	1.00	-	18 (2.5)	1.00	-
Under the diploma	131 (17.9)	145 (19.9)	1.90 (0.87–4.16)	0.108	86 (11.8)	0.96 (0.43–2.14)	0.931
Diploma	81 (11.1)	70 (9.6)	1.54 (0.64–3.68)	0.329	60 (8.2)	1.12 (0.46–2.73)	0.797
College	37 (5.1)	35 (4.8)	2.20 (0.75–6.43)	0.150	28 (3.8)	1.24 (0.40–3.80)	0.708
**Father’s job^a^**							
Unemployed^b^	6 (0.8)	8 (1.1)	1.00	-	5 (0.7)	1.00	-
Worker	43 (5.9)	37 (5.1)	0.63 (0.19–2.07)	0.448	21 (2.9)	0.67 (0.17–2.63)	0.566
Self-employed	125 (17.1)	153 (21)	0.87 (0.28–2.77)	0.822	109 (14.9)	1.48 (0.40–5.50)	0.555
Employee	65 (8.9)	48 (6.6)	0.54 (0.16–1.85)	0.326	35 (4.8)	0.74 (0.18–3.02)	0.676
Retired	33 (4.5)	20 (2.7)	0.46 (0.13–1.64)	0.235	22 (3)	0.88 (0.21–3.66)	0.869
**Mother’s job^a^**							
Housewife^b^	247 (33.8)	246 (33.7)	1.00	-	178 (24.4)	1.00	-
Employed	25 (3. 4)	20 (2.7)	0.85 (0.39–1.81)	0.671	14 (1.9)	0.76 (0.32–1.78)	0.535
**Own room^a^**							
No^b^	76 (10.4)	67 (9.2)	1.00	-	50 (6.8)	1.00	-
Yes	196 (26.8)	199 (27.3)	1.17 (0.77–1.76)	0.454	142 (19.5)	1.19 (0.75–1.90)	0.445
**Living (with)^a^**							
Others^b^	6 (0.8)	14 (1.9)	1.00	-	12 (1.6)	1.00	-
Both parents	261 (35.8)	252 (34.5)	0.64(0.27–1.49)	0.304	180 (24.7)	0.46 (0.19–1.12)	0.090

Reference category: Never WPS. WPS: waterpipe smoking, CI: confidence interval, AOR: adjusted odds ratio.

a Categorical variables

b Reference group

* p<0.05

** p < 0.01

***p < 0.001

Table 4 shows that using Internet more than 7 hours per day (AOR=4.53, 95% CI: 1.82–11.29), ever cigarette smoking (AOR=9.92, 95% CI: 4.68–21.02), current cigarette smoking (AOR=8.41, 95% CI: 2.80–25.48) and family WPS (AOR=7.22, 95% CI: 3.75–13.87) were statistically significant variables in relation to current WPS. Students who used the Internet more than 7 hours a day were 4.53 times more likely to be current smokers of WP compared to those who did not use the Internet and had never smoked WP (p=0.001). There were also significant differences according to ever smoking cigarettes, currently smoking cigarettes and family member smoking WP. These were, respectively, 9.92, 8.41 and 7.22 times more likely to be currently smoking WP than never WPS (all p<0.001).

**Table 4:** Multinomial logistic regression: WPS and behavioral risk factors (n=730)

*Characteristics*	*Never WPS (n=272) (%)*	*Former WPS (n=266) (%)*	*AOR ( 95% CI)*	*p*	*Current WPS (n=192) (%)*	*AOR ( 95% CI)*	*p*
**Computer games^a^**				0.515			0.000
At all^b^	86 (11.8)	80 (11)	1.00	-	62 (85)	1.00	-
Low (1–3 h/d)	84 (11.5)	95 (13)	1.16 (0.73–1.84)	0.516	47 (6.4)	0.65 (0.35–1.19)	0.169
Moderate (3–7 h/d)	58 (7.9)	61 (8.4)	1.07 (0.64–1.79)	0.784	40 (5.5)	0.67 (0.35–1.29)	0.237
High (> 7h/d)	44 (6.0)	30 (4.1)	0.64 (0.35–1.18)	0.159	43 (5.4)	0.58 (0.28–1.18)	0.136
**Internet usage^a^**							
At all^b^	37 (5.1)	29 (4)	1.00	-	9 (1.2)	1.00	-
Low (1–3 h/d)	77 (10.5)	73 (10)	1.00 (0.54–1.88)	0.979	22 (3)	0.98 (0.36–2.67)	0.970
Moderate (3–7 h/d)	73 (10.0)	75 (10.3)	1.08 (0.58–2.04)	0.791	41 (5.6)	2.12 (0.81–5.52)	0.124
High (> 7h/d)	85 (11.6)	89 (12.2)	1.20 (0.65–2.25)	0.550	120 (16.4)	4.53 (1.82–11.29)	0.001***
**Ever cigarette smoking^a^**							
No^b^	258 (35.3)	197 (27)	1.00	-	76 (10.4)	1.00	-
Yes	14 (1.9)	69 (9.5)	5.14 (2.56–10.32)	0.000***	116 (15.9)	9.92 (4.68–21.02)	0.000***
**Current cigarette smoking^a^**
No^b^	267 (36.6)	239 (32.7)	1.00	-	127 (17.4)	1.00	-
Yes	5 (0.7)	27 (3.7)	2.27 (0.74–6.97)	0.151	65 (8.9)	8.41 (2.80–25.48)	0.000***
**WP user friends^a^**							
No^b^	134 (18.4)	177 (24.2)	1.00	-	92 (12.6)	1.00	-
Yes	138 (18.9)	89 (12.2)	0.50 (0.35–0.72)	0.000***	100 (13.7)	0.88 (0.55–1.40)	0.599
**WP user Family^a^**							
No^b^	254 (34.8)	225 (30.8)	1.00	-	134 (18.4)	1.00	-
Yes	18 (2.5)	41 (5.6)	2.55 (1.40–4.64)	0.002**	58 (7.9)	7.22 (3.75–13.87)	0.000***

Reference category: Never WPS. WPS: waterpipe smoking, CI: confidence interval, AOR: adjusted odds ratio.

a Categorical variables

b Reference group

*p<0.05

** p < 0.01

*** p < 0.001

Table 4 also shows that ever smoking cigarettes (AOR=5.14, 95% CI: 2.56–10.32), having WPS friends (AOR=0.50, 95% CI: 0.35–0.72) and having a WPS family member (AOR=2.55, 95% CI: 1.40–4.64) were significant variables in relation to former WPS. Based on this analysis, there were significant differences according to ever smoking cigarettes and having a WPS family member. These were, respectively, 5.14 and 2.55 times more likely to be former smokers of WP compared to never WPS. The odds of former WPS were also negatively associated with having friends who smoked WP; students who had friends that smoked WP were 0.50 times less likely to be former smokers of WP than those whose friends did not smoke WP (p<0.001).

## DISCUSSION

The objective of the present study was to determine the role of sociodemographic factors associated with WPS among male adolescents in Hamadan city. This study has shown that former WPS (36.4%) and current WPS prevalence (26.3%) was quite high among high school students in Hamadan. These findings are in congruence with other studies^9,16^. Prevalence estimates from our study are relatively low compared with studies carried out in some other Islamic countries. For instance, the prevalence of former WPS and current WPS among university students of Jordan was 61.1% and 42.7%, respectively^17^, while ever WPS prevalence was 44.3% and current WPS prevalence was 22.1% in Lebanon^18^.

In the present study, the age range of the first experience with WPS was between 6 and 18 years, with a mean age of 13.31 years. The results of this part of the study were consistent with those of similar studies. For example in Iran, the mean ages for initiation of WPS that were found ranged from 13.39 to 13.80 years^9,19,20^. The results of our study revealed that there is a need to plan and implement effective interventions to prevent tendencies for substance abuse in adolescence.

According to our findings, WPS was associated with the age of students. In this regard, increased age of students added to the chance of WPS. These findings are in congruence with other studies on this topic^9,18,21-24^. It seems that early WPS at a younger age leads to increased addiction to WPS in older ages, resulting in students continuing WPS. Our observation that studying in technical fields resulted in higher odds of WPS than those in the human sciences might indicate differences in understanding of the health risks of hookah smoking and different social norms across disciplines. Similar findings have also been reported elsewhere^25^.

This study has shown that students who use Internet more than 7 hours a day were likely to be current WPS. Studies have shown that Internet addiction is related to tobacco smoking^26,27^. Those addicted to the Internet may conceivably be bored, tired, shy, depressed and suffer from other types of addiction, such as cigarette smoking^28^. Students that reported lifetime (ever) cigarette smoking were significantly more likely to also report former and current WPS than non-smokers of cigarettes. These findings are consistent with previous research^9,29-32^. A possible explanation might be that WPS is being used by youth as an alternative to cigarette smoking among those who want to quit cigarette smoking; it could also be that WPS leads to other forms of tobacco smoking. Also, we found that current cigarette smokers were significantly more likely to be current WPS than others. The results of this part of the study were consistent with the findings of similar studies^24,33^. Perceiving WP as less harmful than cigarettes was positively associated with its use, suggesting that students may be uncertain about the harms associated with WPS relative to cigarette smoking. This misperception was more prevalent among young adults and in never smokers.

Although, previous studies^7,10,31,34^ have shown that having friends as users of WP is related to WPS, our study found that having friends as users of WP was inversely associated with former WPS. The reason for this might be that students who have used WP in the past have had unpleasant health experiences of the negative effects of WPS. The sharing of this unpleasant experience with friends who have not yet smoked hookah might have played a protective role in preventing initiation.

Results from our study show that social influence from family was significantly associated with students’ former and current WPS. There are some studies with findings that are similar to our result^10,35,36^. Family members might own a WP for home use^37^ and hold social gatherings at home to smoke WP^38^. In order to reduce WPS prevalence, public health interventions need not only to target adolescents but also their family members’ risk perceptions, attitudes and behaviors regarding WPS.

This study has some limitations. First, the cross-sectional design used in this study is restricted in establishing cause-effect relationships. Being cross-sectional it provides only a snap-shot in time and does not provide guidance on time sequence. Second, this study was restricted to students in Grades 10–12 in high schools of Hamadan, which limits its generalizability. Third, self-reported data could be prone to bias in reporting (i.e. under reporting or over reporting). Ideally, biochemical testing should have been done, but our results were not biochemically confirmed. Fourth, because of challenges getting permission to enter the girls’ high schools, the current study was restricted to only male high schools. These findings may therefore not apply to all Iranian adolescents.

## CONCLUSIONS

Our study shows that WPS prevalence is high among male high school students in Hamadan. There is a need to design interventional studies to increase students’, friends’ and parents’ knowledge about the harms of WPS and cigarette smoking, to encourage behavior change in this regard, and to put in place restrictive policies for curtailing adolescents’ access to WP and tobacco. In addition, our findings require public health campaigns to access and educate students, teachers and schools, to inform them of the growing understanding of the health effects of WPS.
